# Effect of Pediatric Physical Therapy Interventions on Congenital Muscular Torticollis: A Systematic Review

**DOI:** 10.7759/cureus.69572

**Published:** 2024-09-17

**Authors:** Sakshi Desai, H V Sharath

**Affiliations:** 1 Department of Pediatric Physiotherapy, Ravi Nair Physiotherapy College, Datta Meghe Institute of Higher Education and Research, Wardha, IND

**Keywords:** congenital muscular torticollis, infants, pediatric physiotherapy, physical therapy, rehabilitation

## Abstract

This review explores the current pediatric physiotherapy approaches for treating infants with congenital muscular torticollis (CMT). CMT is a common musculoskeletal condition characterized by an unequal position of the neck due to unilateral shortening of the sternocleidomastoid muscle. Our comprehensive literature search assessed articles published between 2014 and March 2024. The review emphasizes various pediatric physical therapy interventions, such as manual therapy, stretching, soft tissue mobilization techniques, kinesio taping, and microcurrent therapy, along with parental education. The effectiveness of these interventions was assessed in terms of improving the cervical range of motion, reducing muscle tightness, correcting head posture, and improving functional outcomes in infants with CMT. The review underlines the need for early diagnosis and prompt initiation of physiotherapy interventions in order to improve treatment outcomes and reduce long-term musculoskeletal complications associated with CMT. Future research directions are also highlighted to improve our understanding and treatment of this common pediatric condition.

## Introduction and background

Congenital muscular torticollis (CMT) is a condition that normally appears shortly after birth and is defined by an involuntary and asymmetrical head posture, due to the unilateral shortening of the sternocleidomastoid (SCM) muscle, which is frequently accompanied by a fibrotic mass. Ultrasound scans can detect muscle shortening and identify a unique morphological abnormality. It can occur during fetal development or as a result of trauma at the moment of delivery. Fibrotic alterations cause SCM shortening, reducing mobility in the cervical spine area [1-3].

Type 1 CMT is caused by fetal malposition during pregnancy. This disorder is frequently attributed to the unusual placement of the embryonic head and neck, resulting in selective injury to one of the SCM muscles [4,5]. Localized compression or ischemia can induce muscle degeneration. Fetal malposition can be exacerbated by factors such as lack of intrauterine space and inadequate amniotic fluid. This type of CMT is usually seen in infants born breech or in primigravida with insufficient amniotic fluid and excessive intrauterine pressure. Ischemia during fetal development causes muscular edema and gradual degenerative alterations, which may eventually lead to muscle fibrosis [6,7]. Type 2 CMT is caused by prenatal damage, which frequently occurs during breech births or when the fetus's size does not match the delivery canal. This type of injury can result from head tilt or severe twisting during birth, causing SCM muscle fiber rupture and hematoma development that later becomes fibrotic [8-10].

Early management is critical for successfully managing CMT and lowering the risk of long-term problems. Pediatric physical therapy is important to conservative treatment techniques because it emphasizes muscular stretching, strengthening activities, and symmetrical movement patterns. These therapy techniques aim to rectify aberrant head and neck alignment, increase the range of motion (ROM), and promote the child's general growth [11].

Although physical therapy is widely utilized, there is considerable variety in the methods used and the claimed outcomes. Several treatments have been examined, including passive stretching, active exercises, manual therapy, and positional strategies. However, the data on their efficacy and best practices are conflicting. This heterogeneity emphasizes the need for a thorough examination of current procedures and their impact. This narrative review seeks to compile known evidence on pediatric physical therapy interventions for CMT. The review's goal is to provide comprehensive knowledge of the success of various therapeutic techniques by reviewing data from numerous randomized controlled trials (RCTs). It will evaluate the effectiveness of various therapies, their timing, intensity, and effects on both short- and long-term results.

In addition, the review will identify gaps in current research, highlight regions with insufficient or confounding information, and suggest future research study options. Through this comprehensive study, the review hopes to provide significant insights for clinicians and academics, and eventually improve care techniques and outcomes for infants with CMT.

## Review

Methodology

We conducted a systematic review of the literature on pediatric physiotherapy for CMT, focusing on both RCTs and experimental research. The search was performed using Google Scholar, PubMed, and MEDLINE databases, covering publications from January 2014 to March 2024. Only articles published in English were considered for inclusion.

Search strategy

We used specific keywords such as "congenital muscular torticollis," "physiotherapy," and "physical therapy" to identify relevant articles. Boolean operators (AND, OR) were utilized to refine the search results, ensuring a comprehensive collection of studies addressing the research topic.

Inclusion and exclusion criteria

Studies were included if they (1) involved pediatric patients diagnosed with CMT, (2) assessed the effectiveness of physiotherapy interventions, and (3) were either RCTs or experimental research.

Studies were excluded if they (1) were not available in full text, (2) were not peer-reviewed, (3) did not focus on physiotherapy interventions, or (4) were case reports, reviews, or meta-analyses.

Study selection

**Figure 1:**
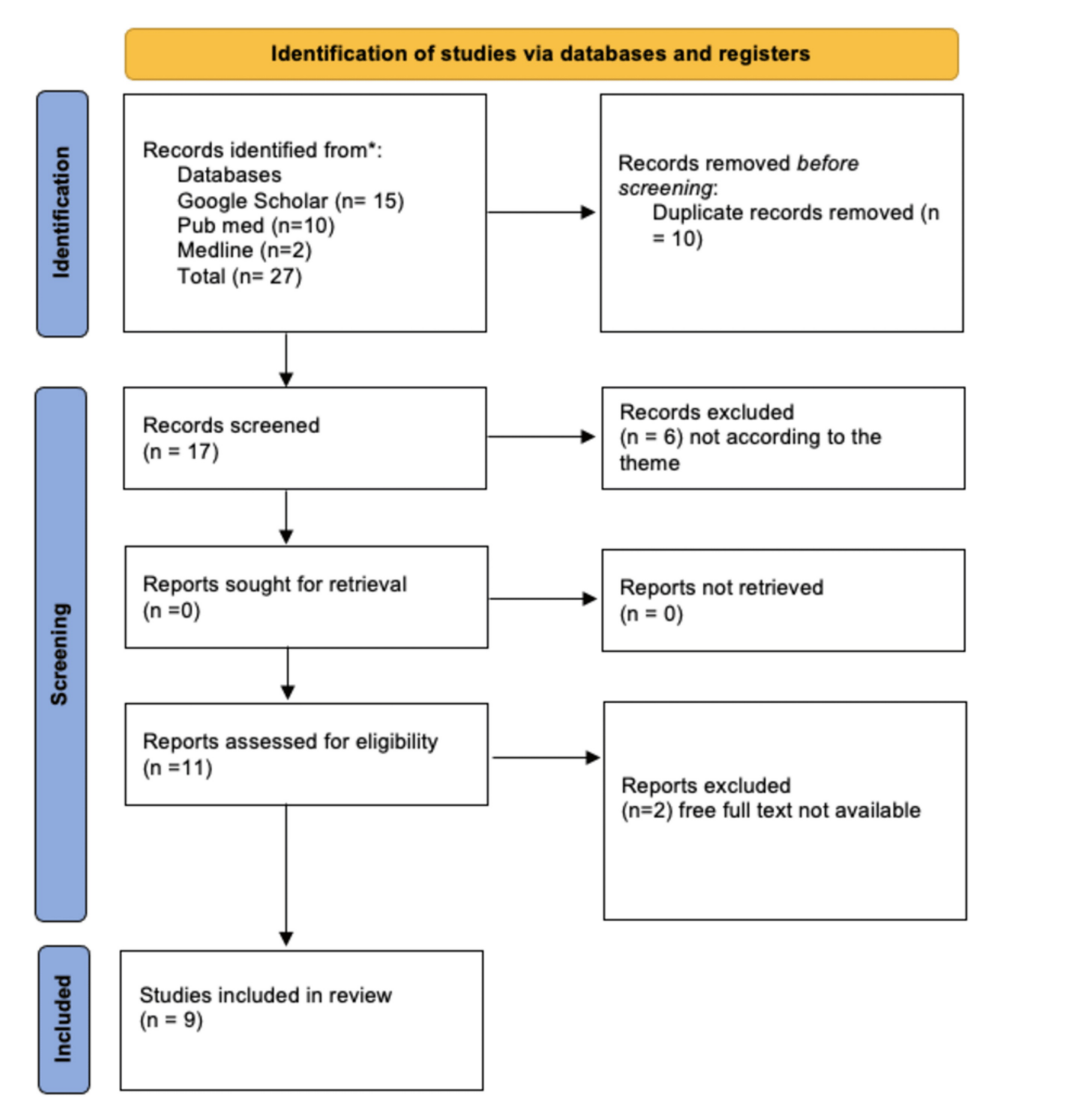
Preferred Reporting Items for Systematic Reviews and Meta-Analyses (PRISMA) flow chart

Initially, 27 articles were identified through the keyword search. After eliminating duplicates and screening the titles and abstracts for relevance, 17 articles were retained. Of these, six articles were excluded, as they did not align with the theme, and two were removed due to a lack of full-text availability. The remaining articles were then reviewed against the inclusion and exclusion criteria, resulting in nine articles being included in the final analysis. The selection process is depicted in Figure 1.

Data extraction and analysis

Key data from the selected studies were extracted, including study design, sample size, intervention details, outcomes measured, and results. A narrative synthesis was performed to summarize the findings, highlighting the efficacy of different physiotherapy interventions for CMT.

Quality assessment

Each included study was assessed for methodological quality using criteria such as randomization, blinding, sample size, and completeness of follow-up. This assessment helped ensure that the conclusions drawn from the review were based on high-quality evidence.

The list of examined studies, which summarize the present landscape of pediatric physical therapy treatment, is mentioned in Table 1.

**Table 1:** Summary of reviewed articles

Sr. No.	Author and date	Participation	Study setting	Inclusion	Outcome measures	Conclusion
1.	Song et al. (2021) [12]	Infants aged between 0 and 3 months	Rehabilitation center in Yongin City in Korea	Infants under three months old have been identified by a medical practitioner with congenital muscular torticollis and demonstrate a head tilt of more than 15 degrees.	Sternocleidomastoid muscle thickness, the ratio of sternocleidomastoid (SCM) muscle thickness between the affected and nonaffected sides (A/N ratio), and the angle of head rotation	Among the interventions studied for increasing the range of motion of head rotation, Passive stretching has been identified as the most effective treatment
2.	Pastor-Pons et al. (2021) [13]	Infants less than 28 weeks	Health Centers in Zaragoza (Spain)	Infants identified by doctors with non-synostotic positional plagiocephaly, aged less than twenty-eight weeks, and a cranial diagonal diameter difference of 5 mm	Alberta infant motor scale (AIMS)	In positional plagiocephaly, combining manual treatment with a caregiver education program has been associated with better neck mobility results. There were no differences in neuromotor development outcomes seen when manual treatment was coupled with a caregiver teaching program in positional plagiocephaly.
3.	Hwang et al. (2019) [14]	0-3 months	Not mentioned	A newborn under three months old, detected during ultrasound testing with a bulge in the sternocleidomastoid muscle, was clinically diagnosed as congenital muscular torticollis (CMT) by a pediatric rehabilitation expert.	Neck ultrasonography	The data suggest that changes in shear wave velocity (SWV) evaluated by acoustic radiation force impulse (ARFI) elastography may serve as a predictor of clinical outcomes, especially the stiffness-related limitation of mobility, in infants undergoing physiotherapy for CMT.
4	Keklicek and Uygur (2018) [15]	Infants between 0 and 6 months of age	Department of Physiotherapy and Rehabilitation Hacettepe University	Head tilt from 5 to 20 degrees	Muscle function scale, degree of head tilt, and range of motion for lateral flexion and rotation of the cervical spine	STM approaches are efficient at producing more rapid improvements in the treatment of CMT.
5	Durguti et al. (2019) [16]	0-9 months of age	Physical Therapy Clinic, Pristina, Kosova	Infants beneath nine months old have a palpable neck mass or limited neck movement.	Cervical lateral flexion range of motion	The early initiation of physical treatment yields far greater benefits.
6.	He et al. (2017) [17]	0-3 months	Department of Rehabilitation, Guangzhou Women and Children Medical Centre	Infants below three months old were evaluated for CMT, defined by head tilt and limited passive neck mobility (lateral flexion and/or rotation), whose parents followed research protocol.	Cervical range of motion, muscle function scale, sternocleidomastoid muscle thickness ratio	Two doses of stretching therapy appear to improve head tilt, passive neck mobility, and sternocleidomastoid muscle development in babies with congenital muscular torticollis. Delivering the therapy 100 times per day may result in significantly improved head tilt and neck mobility.
7	Giray et al. (2017) [18]	3-12 months	Outpatient Pediatric Rehabilitation Clinic	Infants between the age group 3-12 months referred to an outpatient pediatric rehabilitation clinic	Cervical range of motion, muscle function severity scale for assessment of plagiocephaly (PSI)	Kinesiology taping when used in conjunction with therapeutic exercises provides no extra advantages. Different methods of applying kinesiology tape have the same clinical outcomes.
8	Öhman (2015) [19]	2.5 and 12 months	Department of Physiotherapy at Queen Silvia Children’s Hospital, Sweden	Infants with CMT and evident muscle function imbalance in cervical lateral flexors as measured by the muscle function scale (MFS)	Muscle function scale (MFS)	In infants with CMT, applying kinesiology tapping to the affected side immediately affects the MFS associated with muscular imbalance in the cervical lateral flexors.
9	Kwon and Cho (2023) [20]	0-3 months	Not mentioned	The whole sternocleidomastoid muscle is damaged, with an ultrasound-measured thickness of more than 10 mm and a palpable lump inside the muscle.	Passive cervical range of motion, estimated thickness and cross-sectional area of the damaged sternocleidomastoid muscle, red pixel intensity, and length of therapy intervention	Microtherapy in combination with therapeutic exercise and ultrasonography, improves treatment results of CMT affecting the whole SCM muscle.

Intervention

Pastor Pons et al. (2021) proclaimed that the manual treatment program for the upper cervical spine sought to enhance ROM by mobilizing the occiput, atlas, and axes. The procedure entailed placing the infant’s head in the therapist's palms. The fourth and fifth fingers were placed on the occipital bone's condylar region, the middle finger on the axis' articular processes, and the index fingers on the cervical vertebrae beneath C2. The thumbs were placed on the anterior side of the atlas' transverse processes to gently dorsally position. The therapist used myofascial methods to relax cervical myofascial tissues with moderate pressure, while also aiding the child's head movements in flexion, extension, side bending, and rotation, which were consistent with the child's natural motions. Techniques involving end-range placement into cervical extension and rotation were avoided. The individuals in the pediatric integrative manual therapy (PIMT) group received 20-minute treatments once a week for 10 weeks [13].

Keklicek and Uygur's (2018) study applied soft tissue mobilization to reduce tension in the neck muscles and fascia. This method is extensively utilized in clinical settings and can be adapted to various conditions. The intervention consists of three steps. During the "first stage of passive mobilization," the therapist softly but securely grasps the SCM muscle underneath its origin with two or three fingers and rhythmically mobilizes it anteroposteriorly. In the "second phase of mobilization with stretching," the therapist softly holds and slightly extends the muscle, holding this posture before moving it again anteroposteriorly. Finally, in the "third phase," while continuing to gently grasp the muscle, the physical therapist motivates the newborn to execute active cervical rotation towards the affected side by employing vibrant and musical toys to keep the infant's interest [15]. Participants in this trial, proclaimed by He et al. (2017), were subjected to a low-intensity, continuous, pain-free stretching exercise in accordance with CMT treatment recommendations. Each session consisted of 10 manual stretches of the afflicted muscle, each lasting 10 to 15 seconds and separated by a 10-second rest break. Participants varied in their stretching frequency, with some doing 10 sessions per day and others completing just five.

The stretching technique required two therapists: one to retain the baby in a supine position and the other to guide the head through cervical rotation and lateral flexion within a safe range, keeping rotation less than 90 degrees to avoid potential risks [17]. Öhman (2015) stated that Kinesiology taping was adhered with a relaxation method that crossed the afflicted side's SCM muscle. The tape was carefully stretched over the muscular bellies while being applied. The tape stayed on for six to seven minutes until the evaluator returned to test muscular function. This provided adequate time for tape application [19]. Kwon and Cho (2023) administered microcurrent treatment (EMI; Cosmic Co., Seoul, Korea) three times per week, for 30 minutes each session. The treatment used a microcurrent generator configured to provide an alternating current in a monophasic rectangular pulse pattern, switching polarity every three seconds. The frequency and intensity were set at 8 Hz and 200 µA, respectively, which is below newborns' sensory threshold. To enable exact application, the afflicted SCM muscle was separated by rotating the infant's head to the opposite side, allowing for easy palpation and attachment of the electrical patch. After placing the patch, the infant's head was gently restored to its natural posture to avoid straining the SCM muscle during treatment [20], as mentioned in Table 2.

**Table 2:** Interventions used in the treatment of congenital muscular torticollis SCM: Sternocleidomastoid

Technique	Description	Benefits
Manual therapy	Mobilize occiput, atlas, and axis. Gentle pressure, natural motions	Improves range of motion in the upper cervical spine
Soft tissue mobilization	Mobilize SCM muscle with three phases: passive mobilization, mobilization with stretching, active rotation	Reduces tension in neck muscles and fascia
Stretching	Low-intensity stretches, 10-15 seconds each, 10 sessions/day or 5 sessions/day	Improves flexibility and range of motion in the neck
Kinesiology taping	Kinesiology tape was applied across the SCM and held for 6-7 minutes	Provides support and stability to the neck muscles
Microcurrent therapy	Microcurrent applied to SCM 3 times/week, 30 minutes each, 8 Hz, 200 µA	Reduces pain and inflammation in the neck muscles

Discussion

CMT, which affects the neck muscles of the newborn, primarily involves tightness in the SCM muscle, causing the infant to tilt the head to one side. It can lead to developmental delay if left untreated. Early diagnosis and physical therapy interventions are crucial and provide optimal outcomes. A study done by Song et al. (2021) has shown that passive stretching is effective in improving cervical ROM for infants under three months old who are diagnosed with CMT, a condition characterized by cervical muscle stiffness. Other physiotherapeutic interventions, when compared to passive stretching, did not significantly impact the thickness of the SCM muscle.

Passive stretching is superior in enhancing cervical rotation [12]. Pastor Pons et al. (2021) proclaimed that PIMT, along with caregiver education, has been shown to be more effective in improving cervical rotation in infants with positional plagiocephaly. Caregiver education and PIMT optimistically impacted neuromotor development, highlighting the benefits of early intervention for these conditions [13]. A study conducted by Hwang et al. (2019) indicated that the severity of SCM muscle stiffness, measured by acoustic radiation force impulse (ARFI) shear wave velocity (SWV), does not predict long-term outcomes in infants with CMT. Although this measure correlates with initial cervical ROM limitations, it does not show significant changes in SCM tissue properties. While complete neck movements might take longer in cases with higher initial SWV values, more research is required to understand the long-term implications of this measure [14].

Keklicek and Uygur (2018) stated that soft tissue mobilization, when compared to standard care in infants with CMT, has shown short-term benefits in improving neck movement. At the 12-week follow-up, the improvements were not sustained, emphasizing the need for adaptable interventions. These findings highlight the potential of physical therapy interventions for CMT and the requirement for research to support evidence-based practice [15]. Durguti et al. (2019) revealed that early intervention is important in managing CMT. The initiation of physical therapy before the age of six months significantly improves the cervical ROM compared to later interventions. This study sheds light on the importance of timely treatment to optimize neuromotor development and prevent CMT progression [16]. He et al. (2017) explored the impact of stretching on CMT. They found that stretching exercises consistently improved head tilt, neck flexibility, and SCM muscle development in newborns with CMT. The profound benefits of stretching were observed when the frequency was increased, suggesting a dose-response relationship between stretching and treatment outcomes [17].

Giray et al. (2017) found no advantages of kinesiology taping when combined with exercise therapy. Different taping techniques yielded the same clinical outcomes [18]. In contrast, a subsequent study by Öhman (2015), which performed kinesiology taping on the affected side of the neck, observed improvements in muscle function scores after the application of kinesiology tape [19,20]. Kwon and Cho (2023) studied the combined effects of therapeutic exercise, ultrasound, and microcurrent therapy on CMT. This multimodal approach enhanced cervical mobility and reduced the thickness of the SCM muscle compared to ultrasound alone. The study suggests that combining different treatments improves outcomes in infants with CMT. Physical therapy plays a crucial role in managing CMT by addressing muscle imbalances, improving cervical mobility, and enhancing the overall development of the infant. Future research should focus on the development of long-term intervention strategies to improve outcomes for this vulnerable population.

## Conclusions

Pediatric physiotherapy interventions have proven to be highly significant in the treatment of CMT. Early detection and physiotherapeutic intervention are crucial for optimizing treatment outcomes and preventing long-term sequelae. Physical therapy is important in CMT care as it resolves muscular disparities, allows for adequate cervical mobility, and promotes overall developmental growth in infants. Future research should concentrate on treatment tactics for long-term efficacy and improved outcomes for this vulnerable population.
